# Spectrophotometric detection of azole-resistant *Aspergillus fumigatus* with the EUCAST broth microdilution method: is it time for automated MIC reading of EUCAST antifungal susceptibility testing of *Aspergillus* species?

**DOI:** 10.1093/jac/dkac046

**Published:** 2022-02-23

**Authors:** Joseph Meletiadis, Ioanna Efstathiou, Hein A L van der Lee, Karen M T Astvad, Paul E Verweij, Maiken Cavling Arendrup

**Affiliations:** Clinical Microbiology Laboratory, Attikon University Hospital, Athens, Greece; Department of Medical Microbiology and Infectious Diseases, Erasmus Medical Center, Rotterdam, The Netherlands; Clinical Microbiology Laboratory, Attikon University Hospital, Athens, Greece; Department of Medical Microbiology, Radboud University Medical Centre, and Center of Expertise in Mycology Radboudumc/CWZ, Nijmegen, The Netherlands; Unit of Mycology, Statens Serum Institut, Copenhagen, Denmark; Department of Medical Microbiology, Radboud University Medical Centre, and Center of Expertise in Mycology Radboudumc/CWZ, Nijmegen, The Netherlands; Centre for Infectious Diseases Research, Diagnostics and Laboratory Surveillance, National Institute for Public Health and the Environment (RIVM), Bilthoven, The Netherlands; Unit of Mycology, Statens Serum Institut, Copenhagen, Denmark; Department of Clinical Microbiology, Rigshospitalet, Copenhagen, Denmark; Department of Clinical Medicine, University of Copenhagen, Copenhagen, Denmark

## Abstract

**Objectives:**

Current reference susceptibility testing methods of *Aspergillus* require visual reading, which is subjective and necessitates experienced staff. We compared spectrophotometric and visual MIC reading of EUCAST E.Def 9.3.2 susceptibility testing of *Aspergillus fumigatus* for a large collection of isolates with different azole resistance mechanisms.

**Methods:**

*A. fumigatus* (*n* = 200) were examined, including 62 WT and 138 non-WT with the following alterations: TR_34_/L98H (*n* = 57), TR_46_/Y121F/T289A (*n* = 54) or single point mutations (*n* = 27). EUCAST E.Def 9.3.2 susceptibility testing was performed for amphotericin B, itraconazole, voriconazole, posaconazole and isavuconazole. MICs were determined after 48 h of incubation visually and spectrophotometrically, as the lowest concentration corresponding to a 1%, 3%, 5%, 10% or 15% OD increase above the background OD. The best spectrophotometric endpoint (SPE) was identified based on the highest essential agreement (EA; ±1 two-fold dilution) and categorical agreement (CA) and fewer very major errors (VMEs) and major errors (MEs).

**Results:**

Τhe best SPEs were 5% and 10% for all drugs. The best agreement between visual and spectrophotometric MICs was found with the 10% growth endpoint, which resulted in identical median MICs with 90% of differences being ≤1 two-fold and higher EA (91%–100%) and CA (100%) and no VMEs and MEs compared with the 5% endpoint (77%–100%, 96%–98%, 0% and 0%–4%, respectively).

**Conclusions:**

Spectrophotometric MIC reading can be used for *A. fumigatus* susceptibility testing and for detecting azole resistance. A visual inspection of the plate should be performed to confirm equal inoculation, absence of well contamination and proper growth, and to identify potential uncommon phenotypes or subpopulations.

## Introduction

Azole resistance in *Aspergillus fumigatus* is rising and associated with a significantly increased risk of therapeutic failure, making its detection of paramount importance for early change of antifungal therapy.^1^ EUCAST^2^ and CLSI^3^ developed reference methods for the determination of MICs for clinically relevant moulds. These methods are microbroth dilution methods and based on visual reading of growth inhibition. In contrast, EUCAST methodologies for antifungal susceptibility testing of yeasts and dermatophytes rely on spectrophotometric reading, which increases objectivity and facilitates automation, thereby avoiding human errors associated with visual MIC reading. We evaluated spectrophotometric reading for the determination of MICs of antifungal azoles for *A. fumigatus* with EUCAST E.Def 9.3.2 with very promising results.^4^ Since few resistant isolates with limited MIC ranges were included in that study, we have now evaluated the spectrophotometric reading of EUCAST E.Def 9.3.2 for 200 clinical isolates, including a large panel of isolates harbouring resistance mechanisms associated with the *Cyp51A* target gene. The resistance mutations included TR_34_/L98H, TR_46_/Y121F/T289A and various single point mutations. Together the included mutants covered the most common resistance mutations found in clinical azole-resistant *A. fumigatus* isolates.

## Methods

This study included the following 200 clinical *A. fumigatus* isolates (different from those tested in Meletiadis *et al*.^4^): 62 isolates with a WT phenotype for azoles (based on MICs and sequencing) and amphotericin B (based only on MICs), 57 isolates harbouring TR_34_/L98H, 54 isolates harbouring TR_46_/Y121F/T289A of which 19 isolates had one to three additional single point mutations (G448S, R358G, S363P, I364V, M172I or V119A; Nijmegen culture collection, 2018–20, molecularly identified and *Cyp51A* sequenced^5^) and 27 isolates harbouring G54R/W (7), P216S (3), G448S (3), G432S (1), M220K/I/R (9), Y121F (2), F219S (1) or TR_120_/F46Y/M172V/E427K (1) (SSI culture collection, 2015–20, molecularly identified and *Cyp51A* sequenced^5^). Overall, 173 *A. fumigatus* isolates (62 WT and 111 non-WT isolates) were tested in Nijmegen and 27 *A. fumigatus* isolates (0 WT and 27 non-WT isolates) were tested in Copenhagen (Table S1, available as Supplementary data at *JAC* Online).

Susceptibility to amphotericin B, itraconazole, voriconazole, posaconazole and isavuconazole was determined once and read both visually and spectrophotometrically in the two centres (in Denmark and in the Netherlands) following EUCAST E.Def 9.3.2 methodology and analysed retrospectively. Isolates were stored in 10% glycerol (SSI Diagnostica, Hillerød in Denmark and MediaProducts in the Netherlands) at −80°C, subcultured twice on Sabouraud glucose agar at 35°C for 5–7 days in order to revive and harvested using sterile water with 0.1% Tween 20. Conidial suspensions were then filtered using 11 μm nylon filters (Millipore, Tullagreen Carrigtwohill, Ireland) (in Denmark) or the suspension was left to stand for a few minutes to allow heavy particles to settle (in the Netherlands). The concentration was adjusted using a spectrophotometer to obtain twice the final inoculum of 1 × 10^5^ to 2.5 × 10^5^ cfu/mL according to EUCAST E.Def 9.3.2 and using 96-well flat-bottom microtitre trays (NUNC 167008, ThermoFisher, Waltham, MA, USA in Denmark and Clear Flat Bottom Polystyrene TC-treated Microplate, Corning in the Netherlands). Pure compounds of amphotericin B, posaconazole and itraconazole (Sigma–Aldrich), voriconazole (Pfizer) and isavuconazole (Basilea Pharmaceutica Ltd) were dissolved in DMSO (Sigma–Aldrich) and serially diluted in the microplates in double-concentrated EUCAST growth medium (in Denmark) or using the ISO standard with stepwise dilutions in DMSO and then in double-concentrated EUCAST growth medium as described in EUCAST E.Def 9.3.2^2^ (in the Netherlands) to yield final concentrations within the range 0.004–16 mg/L for amphotericin B, itraconazole, voriconazole, posaconazole and isavuconazole. Trays were kept at −80°C until the day of testing. On the day of the experiment the microtitration trays were defrosted, inoculated with 100 μL of inoculum and incubated at 37°C for 2 days.

The MICs of the antifungal drugs were determined visually as the lowest concentration resulting in no visible growth according to EUCAST E.Def 9.3.2 and spectrophotometrically (EL808 Ultra Microplate Reader, BIO-TEK instruments, Inc., Holm & Halby in Denmark and Anthos HT3, Biochrome, Germany in the Netherlands) with a reading at a single point in the middle of the well at 490 nm in Denmark or at 35 points all over the well at 405 nm in the Netherlands. After subtraction of the ODs of the mock inoculated negative control wells, the percentage of growth for each well was calculated with the following equation: (OD_test well_ − mean OD_neg control_)/(OD_pos control_ − mean OD_neg control_) × 100. The spectrophotometric endpoint (SPE) MIC was determined as the lowest concentration corresponding to a 1%, 3%, 5%, 8%, 10% or 15% OD increase above the background OD. Single wells with growth at the highest concentration followed by wells with no growth at lower concentrations were ignored.

The median (range) and 90th percentile (MIC_90_) visual and SPE MICs were determined for each compound for WT and non-WT isolates. The median (range) differences and the 90th percentile of numeric differences (absolute values of differences) between each SPE and visual MICs were calculated. The essential agreement (EA; ±1 two-fold dilution) and categorical agreement (CA; susceptible/resistant) between visual and SPE readings were calculated for each SPE. The recently revised EUCAST breakpoints were used for the classification of isolates as susceptible/resistant: ≤1/>1 mg/L for amphotericin B, ≤1/>1 mg/L for itraconazole and voriconazole, ≤0.125/>0.25 mg/L for posaconazole [isolates with a posaconazole MIC at the newly introduced area of technical uncertainty (ATU) of 0.25 mg/L were considered susceptible to posaconazole if they were susceptible to itraconazole; otherwise they were considered resistant to posaconazole] and ≤1/>2 mg/L for isavuconazole (isolates with an isavuconazole MIC at the ATU of 2 mg/L were considered susceptible to isavuconazole if they were susceptible to voriconazole; otherwise they were considered resistant to isavuconazole).^6^

The visual endpoint was used as the gold standard. If the two methods classified isolates identically it was considered as CA. Major errors (MEs) were considered when spectrophotometric MIC readings classified an isolate as resistant and the visual MIC readings classified the isolate as susceptible. Very major errors (VMEs) were considered when spectrophotometric MIC readings classified an isolate as susceptible and the visual MIC readings classified the isolate as resistant. Since there is no I category (susceptible increased exposure) for *A. fumigatus*, no minor error (mE) rates were determined. The analysis was performed for each group of isolates (WT, TR_34_/L98H, TR_46_/Y121F/T289A and single point mutations) separately. The SPE with the highest CA and fewer VMEs and MEs was defined as the most optimal.

## Results

**Figure 1.**
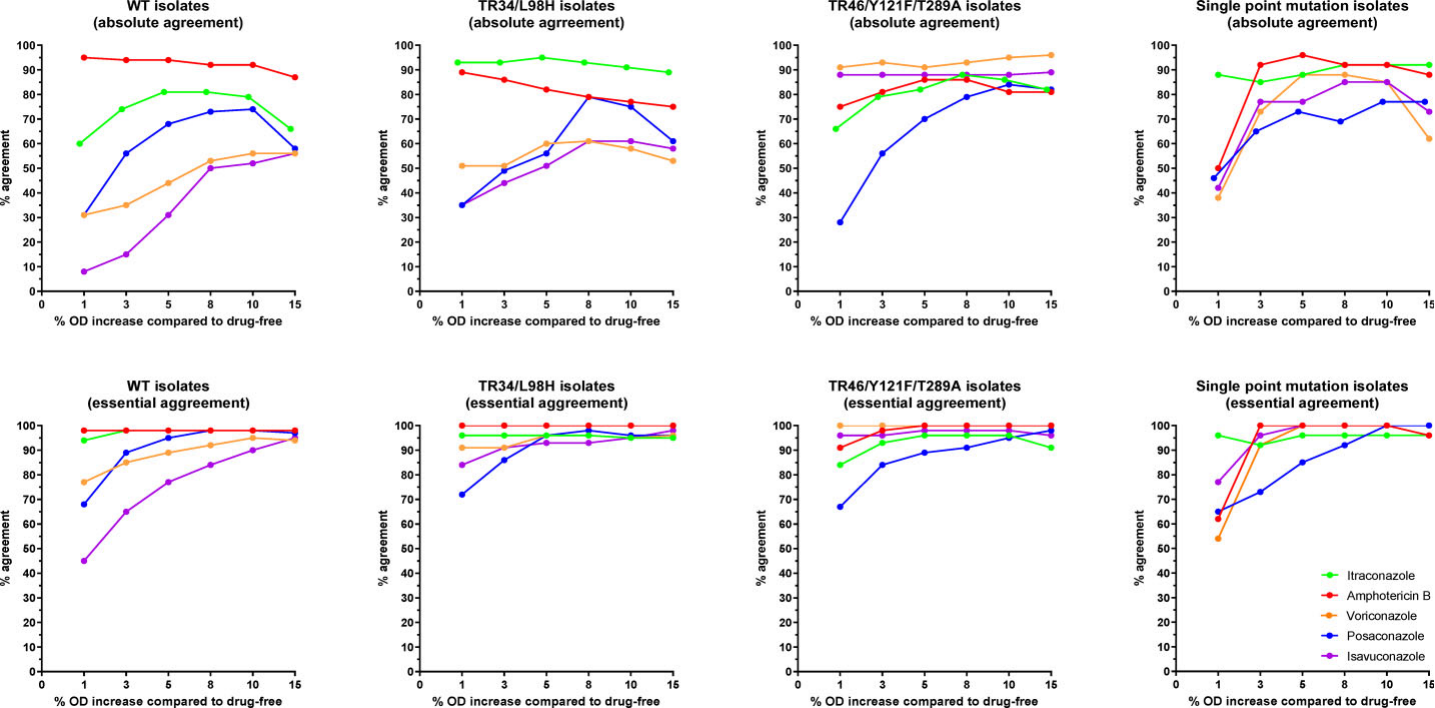
Absolute agreement (no difference) and EA (within 1 two-fold dilution) between visual and spectrophotometric readings of EUCAST E.Def 9.3.2 antifungal susceptibility testing of *A. fumigatus* for each drug and group of isolates. This figure appears in colour in the online version of *JAC* and in black and white in the print version of *JAC*.

Across all compounds, the best EA between visual and spectrophotometric MICs was found when adopting growth endpoints in the 5%–10% range to determine the spectrophotometric MICs compared with 1%, 3% or 15% growth (Figure 1). The median (range) MICs and differences between the two methods using 5% and 10% growth as endpoints are shown in Table 1 for each of the five antifungals, individually. The median differences were 0 two-fold dilutions for all agents and isolates with the 10% endpoint, but not for the 5% endpoint and isavuconazole against WT isolates (1 two-fold dilution difference). The 90th percentile of numeric differences was ≤1 two-fold dilution for all agents and isolates with the 10% endpoint, but for the 5% endpoint this was not the case for voriconazole/isavuconazole against the WT isolates and for posaconazole against isolates with single point mutations (2 two-fold dilution differences). Most of the differences were within 1 two-fold dilution (77%–100% for the 5% endpoint and 91%–100% for the 10% endpoint). The CA ranged from 99% to 100% with 0% VMEs and 0%–4% MEs for the 5% endpoint; values for the 10% endpoint were 100%, 0% and 0%, respectively. MEs were only observed with the 5% endpoint and were found for itraconazole/posaconazole against one WT isolate and voriconazole against one isolate that harboured a single point mutation. In detail, the itraconazole/posaconazole MICs for the WT isolate were 1/0.25 mg/L with visual reading resulting in classification of S/S compared with 2/0.25 mg/L based on the 5% SPE resulting in classification of R/R (posaconazole ATU interpreted based on itraconazole susceptibility) and 1/0.125 mg/L based on the 10% SPE resulting in classification of S/S. For the isolate with the single point mutation P216S, the voriconazole visual MIC was 1 mg/L and was susceptible to all azoles and had spectrophotometric MICs of 2 and 0.5 mg/L based on the 5% endpoint and the 10% endpoint, respectively.

**Table 1.** Comparison between visual (complete growth inhibition) and different endpoints of spectrophotometric MIC readings of EUCAST E.Def.9.3.2 antifungal susceptibility testing of *A. fumigatus* against amphotericin B and azoles

Endpoint	Isolates	Agent	Visual median (range)/MIC_90_	Spectrophotometric median (range)/MIC_90_	Number of two-fold differences, median (range)/90% of numerical values	Percentage of agreement within two-fold dilutions			Percentage of CA and errors		
						0	1	2	CA	VMEs	MEs
5% growth	WT (*n* = 62)	AMB	0.5 (0.25 to 1)/1	0.5 (0.25 to 1)/1	0 (0 to 1)/0	95	100	100	100	0	0
		ITC	0.25 (0.063 to 2)/0.5	0.25 (0.063 to 2)/0.5	0 (−1 to 1)/1	81	100	100	98	0	2
		VRC	0.5 (0.125 to 1)/0.5	0.5 (0.25 to 1)/1	0 (−2 to 1)/2	47	89	100	100	0	0
		POS	0.063 (0.031 to 0.25)/0.125	0.063 (0.031 to 0.25)/0.25	0 (−2 to 1)/1	69	95	100	98	0	2
		ISA	0.25 (0.125 to 4)/1	0.5 (0.25 to 4)/1	−1 (−3 to 1)/2	32	77	96	100	0	0
									
	TR_34_/L98H (*n* = 57)	AMB	0.5 (0.25 to 1)/1	0.5 (0.125 to 1)/1	0 (−1 to 1)/1	86	100	100	100	0	0
		ITC	>16 (2 to >16)/>16	>16 (>16 to >16)/>16	0 (−4 to 0)/0	95	97	97	100	0	0
		VRC	4 (2 to 16)/8	4 (2 to 16)/8	0 (−2 to 1)/1	60	97	100	100	0	0
		POS	0.5 (0.125 to 1)/0.5	0.5 (0.125 to 2)/1	0 (−2 to 1)/1	56	96	100	100	0	0
		ISA	8 (2 to >8)/>8	8 (4 to >8)/>8	0 (−2 to 1)/1	51	93	100	100	0	0
									
	TR_46_/Y121F/T289A (*n* = 54)	AMB	0.5 (0.125 to 1)/1	0.5 (0.125 to 1)/1	0 (−1 to 1)/1	83	100	100	100	0	0
		ITC	2 (0.25 to >16)/>16	4 (0.25 to >16)/>16	0 (−3 to 1)/0.7	89	96	98	100	0	0
		VRC	>16 (0.5 to >16)/>16	>16 (0.5 to >16)/>16	0 (−1 to 1)/0	91	100	100	100	0	0
		POS	0.5 (0.016 to 16)/1	1 (0.016 to 16)/2	0 (−3 to 0)/1	78	93	99	100	0	0
		ISA	>8 (0.5 to >8)/>8	>8 (1 to >8)/>8	0 (−1 to 0)/0.7	89	100	100	100	0	0
									
	single point mutations (*n* = 27)	AMB	0.5 (0.125 to 2)/1	0.5 (0.125 to 2)/1	0 (0 to 1)/0	96	100	100	100	0	0
		ITC	>16 (0.5 to >16)/>16	>16 (0.5 to >16)/>16	0 (−1 to 3)/0.4	88	96	100	100	0	0
		VRC	1.5 (0.5 to >16)/11.2	2 (0.25 to >16)/11.2	0 (0 to 1)/0.4	88	100	100	96	0	4
		POS	2 (0.125 to 8)/8	2 (0.125 to 8)/8	0 (−4 to 1)/2	73	85	97	100	0	0
		ISA	2 (0.5 to >8)/>8	2 (0.5 to >8)/>8	0 (−1 to 1)/1	77	100	100	100	0	0
									
10% growth	WT (*n* = 62)	AMB	0.5 (0.25 to 1)/1	0.5 (0.125 to 1)/1	0 (0 to 1)/0	94	100	100	100	0	0
		ITC	0.25 (0.063 to 2)/0.5	0.25 (0.063 to 2)/0.5	0 (−1 to 1)/1	81	100	100	100	0	0
		VRC	0.5 (0.125 to 1)/0.5	0.25 (0.25 to 1)/0.5	0 (−2 to 2)/1	56	93	99	100	0	0
		POS	0.063 (0.031 to 0.25)/0.125	0.063 (0.016 to 0.25)/0.125	0 (−2 to 1)/1	73	99	100	100	0	0
		ISA	0.25 (0.125 to 4)/1	0.5 (0.25 to 4)/1	0 (−2 to 1)/1	52	91	100	100	0	0
									
	TR_34_/L98H (*n* = 57)	AMB	0.5 (0.25 to 1)/1	0.5 (0.125 to 1)/1	0 (−1 to 1)/1	81	100	100	100	0	0
		ITC	>16 (2 to >16)/>16	>16 (4 to >16)/>16	0 (−4 to 3)/0	91	95	95	100	0	0
		VRC	4 (2 to 16)/8	4 (2 to 8)/8	0 (−2 to 1)/1	61	96	100	100	0	0
		POS	0.5 (0.125 to 1)/0.5	0.5 (0.125 to 1)/1	0 (−2 to 1)/1	81	99	100	100	0	0
		ISA	8 (2 to >8)/>8	8 (4 to >8)/>8	0 (−2 to 1)/1	63	95	100	100	0	0
									
	TR_46_/Y121F/T289A (*n* = 54)	AMB	0.5 (0.125 to 1)/1	>16 (1 to >16)/>16	0 (−1 to 1)/1	80	100	100	100	0	0
		ITC	2 (0.25 to >16)/>16	>16 (1 to >16)/>16	0 (−2 to 4)/1	85	92	94	100	0	0
		VRC	>16 (0.5 to >16)/>16	>16 (1 to >16)/>16	0 (−1 to 1)/0	93	100	100	100	0	0
		POS	0.5 (0.016 to 16)/1	>16 (1 to >16)/>16	0 (−2 to 2)/0.7	89	95	100	100	0	0
		ISA	>8 (0.5 to >8)/>8	>8 (1 to >8)/>8	0 (−1 to 0)/0.7	89	100	100	100	0	0
									
	single point mutations (*n* = 27)	AMB	0.5 (0.125 to 2)/1.7	0.5 (0.125 to 2)/1	0 (0 to 1)/0	92	100	100	100	0	0
		ITC	>16 (0.5 to >16)/>16	>16 (0.5 to >16)/>16	0 (−1 to 4)/0	92	98	100	100	0	0
		VRC	1.5 (0.5 to >16)/12	2 (0.25 to >16)/11.2	0 (0 to 1)/1	85	100	100	100	0	0
		POS	2 (0.125 to 8)/8	2 (0.125 to 8)/8	0 (−1 to 1)/1	77	100	100	100	0	0
		ISA	2 (0.5 to >8)/>8	2 (0.5 to >8)/16	0 (0 to 1)/1	85	100	100	100	0	0

AMB, amphotericin B; ITC, itraconazole; VRC, voriconazole; POS, posaconazole; ISA, isavuconazole.

## Discussion

In this study we show that a spectrophotometric MIC determination of EUCAST antifungal susceptibility testing of *A. fumigatus* isolates with spectrophotometric 10% growth endpoints was in very good to excellent EA (91%–100%) and CA (100%) with the standard visual MIC determination. Of note, EA in mycology is usually set at ±2 two-fold dilutions and therefore an EA within 1 two-fold dilution as set in the present study of >91% is extremely high. No VMEs and MEs were found with the 10% endpoint, whereas 0%–4% MEs were observed with the 5% endpoint, as slight variation in OD of the wells may increase growth just above the 5% endpoint without affecting the 10% endpoint.

Spectrophotometric quantitation of *Aspergillus* growth was not adopted in EUCAST and CLSI reference documents due to a concern that uneven growth might be overlooked due to the non-uniform filamentous growth. Although this is a relevant concern, our data suggest that filamentous growth is sufficiently uniform to be quantitated with a spectrophotometer. Several factors may explain this. Surface growth that may obscure spectrophotometric readings is rare and is minimized with the presence of Tween 20 used for the inoculum preparation and therefore present in the culture broth.^7^ Moreover, hyphal clumps if not removed from the inoculum may contribute to uneven growth. We used an inoculum prepared with filtration or after heavy particles were able to settle down before use, promoting a more homogeneous inoculum and subsequently more even growth in flat-bottom wells. This may be the reason why studies investigating spectrophotometric reading of CLSI M38A MICs used flat-bottom microtitration plates although CLSI normally use round-bottom plates.^8–11^ Even for slow growing isolates with an OD as low as 0.100 above the background OD, spectrophotometric reading was sufficient to determine the MIC reliably. As high agreement was found in both centres, which used different wavelengths for spectrophotometric readings (405 and 490 nm), the wavelength does not seem to be important as long as proper background OD is subtracted. This is in line with recent publications where 540 nm was used and high agreement between visual and spectrophotometric readings was found.^12^ A range of wavelengths spanning from 405 to 530 nm is also recommended in EUCAST E.Def 7.3.2 for antifungal susceptibility testing of yeasts.^13^

VMEs may occur due to uneven growth of resistant isolates, subpopulations of resistant isolates or off-reading axis pinpoint mycelia that are not captured by a spectrophotometer but would be detected by visual inspection. A recent study suggested this is a relevant risk for susceptibility testing of itraconazole and posaconazole against *A. fumigatus* cryptic species and voriconazole against *A. fumigatus* species complex.^14^ Most misclassifications were found for voriconazole and isavuconazole in the ATU or close to the respective breakpoints.^14^ However, no difference was found between the 5% and 10% endpoint for *A. fumigatus sensu stricto* in a recently published study, whereas a 5% endpoint was suggested for *A. fumigatus* cryptic species.^12^ More VMEs were found with the 10% endpoint compared with the 5% endpoint in a previous study using previous EUCAST breakpoints (v 9.0 before implementation of the ATU) and a small collection of resistant isolates, with mainly non-TR mutations in *Cyp51A*.^4^ Despite the fact that a large collection of resistant isolates was tested, no VMEs were observed in the present study using EUCAST breakpoints v 10.0 where ATUs have been introduced. However, we do advise that a visual inspection of plates should be performed before reporting the spectrophotometric MIC, particularly for unusual phenotypes. Such an inspection will also ensure equal volumes in wells, that no artefacts are present and the absence of contaminated wells.

Limitations of the present study were the two-centre nature and the lack of inclusion of resistant isolates with mechanisms other than *Cyp51A* mutations. However, the included mutant isolates covered a wide range of percentage growth near the MIC, which may reflect different patterns of growth also observed with other resistance mechanisms. In addition, earlier incubation timepoints were not evaluated in order to test whether spectrophotometric reading can be used for early detection of resistance.

In conclusion, our data suggest that spectrophotometric determination of azole MICs is an alternative to visual endpoint reading for *A. fumigatus*. When also taking into consideration the potential for increased objectivity, automation and high-throughput testing for EUCAST E.Def 9.3.2 antifungal susceptibility testing, we believe that SPE reading deserves consideration for being incorporated into the reference methodology. Since the 10% endpoint resulted in 100% CA and no VMEs and MEs, we recommend this endpoint, providing that visual inspection is performed before the MIC is reported.

## Supplementary Material

dkac046_Supplementary_Data

## Supplementary data

Table S1 is available as Supplementary data at *JAC* Online.
