# Supplementary material for: Spectrophotometric detection of azole-resistant Aspergillus fumigatus with the EUCAST broth microdilution method: is it time for automated MIC reading of EUCAST antifungal susceptibility testing of Aspergillus species?
Source: J Antimicrob Chemother. 2022 Feb 23;77(5):1296–300. doi: 10.1093/jac/dkac046 (PMC9840474; doi:10.1093/jac/dkac046)
Supplement: dkac046_Supplementary_Data [file dkac046_supplementary_data.docx]

**Supplementary data**

**Table S1.** EUCAST MICs in mg/L

| **N** | **CYP51A** | **AMB** | **ITC** | **VRC** | **POS** | **ISA** |
| --- | --- | --- | --- | --- | --- | --- |
| **1** | WT | 1 | 0.5 | 0.5 | 0.063 | 0.5 |
| **2** | WT | 1 | 0.5 | 0.5 | 0.125 | 1 |
| **3** | WT | 1 | 0.5 | 0.5 | 0.125 | 1 |
| **4** | WT | 1 | 1 | 0.5 | 0.125 | 1 |
| **5** | WT | 1 | 0.5 | 0.25 | 0.063 | 1 |
| **6** | WT | 1 | 0.5 | 0.5 | 0.125 | 1 |
| **7** | WT | 1 | 1 | 0.5 | 0.063 | 1 |
| **8** | WT | 1 | 0.5 | 0.5 | 0.063 | 0.5 |
| **9** | WT | 0.5 | 0.5 | 0.5 | 0.063 | 0.5 |
| **10** | WT | 0.5 | 0.25 | 1 | 0.125 | 0.5 |
| **11** | WT | 0.5 | 2 | 1 | 0.25 | 4 |
| **12** | WT | 0.5 | 1 | 1 | 0.25 | 2 |
| **13** | WT | 0.5 | 0.063 | 0.25 | 0.031 | 0.25 |
| **14** | WT | 1 | 0.5 | 0.5 | 0.125 | 1 |
| **15** | WT | 0.5 | 0.125 | 1 | 0.031 | 0.25 |
| **16** | WT | 0.5 | 0.125 | 0.25 | 0.031 | 0.25 |
| **17** | WT | 0.25 | 0.25 | 0.5 | 0.125 | 0.5 |
| **18** | WT | 0.25 | 0.25 | 0.25 | 0.063 | 0.25 |
| **19** | WT | 0.25 | 0.125 | 0.25 | 0.031 | 0.25 |
| **20** | WT | 0.5 | 0.125 | 0.5 | 0.063 | 0.5 |
| **21** | WT | 0.25 | 0.125 | 0.25 | 0.031 | 0.125 |
| **22** | WT | 0.5 | 0.125 | 0.125 | 0.031 | 0.125 |
| **23** | WT | 0.5 | 0.25 | 0.125 | 0.031 | 0.125 |
| **24** | WT | 0.5 | 0.5 | 0.25 | 0.063 | 0.25 |
| **25** | WT | 0.5 | 0.25 | 0.25 | 0.063 | 0.25 |
| **26** | WT | 0.5 | 0.25 | 0.25 | 0.063 | 0.25 |
| **27** | WT | 0.5 | 0.25 | 0.25 | 0.063 | 0.125 |
| **28** | WT | 0.25 | 0.25 | 0.5 | 0.125 | 0.5 |
| **29** | WT | 0.25 | 0.25 | 1 | 0.063 | 0.5 |
| **30** | WT | 0.25 | 0.125 | 0.25 | 0.031 | 0.25 |
| **31** | WT | 0.5 | 0.125 | 0.5 | 0.031 | 0.25 |
| **32** | WT | 0.5 | 0.25 | 0.5 | 0.063 | 0.5 |
| **33** | WT | 1 | 0.125 | 0.25 | 0.031 | 0.25 |
| **34** | WT | 0.25 | 0.125 | 0.25 | 0.031 | 0.25 |
| **35** | WT | 0.25 | 0.125 | 0.5 | 0.031 | 0.25 |
| **36** | WT | 0.25 | 0.125 | 0.5 | 0.031 | 0.5 |
| **37** | WT | 0.25 | 0.125 | 0.5 | 0.063 | 0.25 |
| **38** | WT | 0.5 | 0.25 | 0.25 | 0.063 | 0.25 |
| **39** | WT | 0.5 | 0.125 | 0.5 | 0.031 | 0.25 |
| **40** | WT | 0.5 | 0.125 | 0.5 | 0.031 | 0.5 |
| **41** | WT | 0.5 | 0.25 | 0.25 | 0.063 | 0.5 |
| **42** | WT | 0.25 | 0.063 | 0.25 | 0.031 | 0.125 |
| **43** | WT | 0.25 | 0.125 | 0.25 | 0.031 | 0.125 |
| **44** | WT | 0.5 | 0.125 | 0.5 | 0.063 | 0.25 |
| **45** | WT | 1 | 0.25 | 0.5 | 0.125 | 0.5 |
| **46** | WT | 0.5 | 0.25 | 0.5 | 0.125 | 0.25 |
| **47** | WT | 1 | 0.25 | 0.25 | 0.063 | 0.25 |
| **48** | WT | 0.5 | 0.125 | 0.25 | 0.063 | 0.25 |
| **49** | WT | 0.5 | 0.063 | 0.25 | 0.031 | 0.25 |
| **50** | WT | 0.5 | 0.125 | 0.25 | 0.031 | 0.25 |
| **51** | WT | 0.5 | 0.125 | 0.5 | 0.031 | 0.25 |
| **52** | WT | 0.5 | 0.25 | 0.5 | 0.063 | 0.25 |
| **53** | WT | 0.25 | 0.5 | 0.5 | 0.125 | 0.5 |
| **54** | WT | 0.5 | 0.25 | 0.25 | 0.063 | 0.25 |
| **55** | WT | 0.25 | 0.125 | 0.25 | 0.031 | 0.125 |
| **56** | WT | 0.25 | 0.125 | 0.25 | 0.031 | 0.125 |
| **57** | WT | 0.25 | 0.125 | 0.125 | 0.031 | 0.125 |
| **58** | WT | 1 | 1 | 1 | 0.25 | 1 |
| **59** | WT | 1 | 0.25 | 0.5 | 0.125 | 0.5 |
| **60** | WT | 0.5 | 0.125 | 0.5 | 0.063 | 0.25 |
| **61** | WT | 0.25 | 0.125 | 0.25 | 0.031 | 0.25 |
| **62** | WT | 0.25 | 0.125 | 0.25 | 0.031 | 0.25 |

| **N** | **CYP51A** | **AMB** | **ITC** | **VRC** | **POS** | **ISA** |
| --- | --- | --- | --- | --- | --- | --- |
| **1** | TR_34_/L98H | 0.25 | >16 | 8 | 0.25 | 8 |
| **2** | TR_34_/L98H | 1 | 2 | 2 | 0.25 | 8 |
| **3** | TR_34_/L98H | 0.25 | >16 | 2 | 0.25 | >8 |
| **4** | TR_34_/L98H | 0.5 | >16 | 8 | 0.5 | >8 |
| **5** | TR_34_/L98H | 0.25 | >16 | 4 | 0.25 | >8 |
| **6** | TR_34_/L98H | 0.25 | >16 | 4 | 0.25 | 8 |
| **7** | TR_34_/L98H | 0.25 | >16 | 2 | 0.5 | >16 |
| **8** | TR_34_/L98H | 1 | >16 | 8 | 1 | 8 |
| **9** | TR_34_/L98H | 0.25 | >16 | 8 | 0.5 | >16 |
| **10** | TR_34_/L98H | 0.25 | >16 | 2 | 0.125 | 8 |
| **11** | TR_34_/L98H | 0.25 | >16 | 4 | 0.25 | 8 |
| **12** | TR_34_/L98H | 1 | >16 | 4 | 0.5 | 8 |
| **13** | TR_34_/L98H | 0.5 | >16 | 2 | 0.25 | 8 |
| **14** | TR_34_/L98H | 0.5 | >16 | 2 | 0.25 | 8 |
| **15** | TR_34_/L98H | 0.5 | >16 | 16 | 0.5 | >8 |
| **16** | TR_34_/L98H | 1 | >16 | 4 | 0.25 | 8 |
| **17** | TR_34_/L98H | 0.25 | >16 | 8 | 0.25 | 8 |
| **18** | TR_34_/L98H | 0.25 | >16 | 8 | 0.5 | >16 |
| **19** | TR_34_/L98H | 0.5 | >16 | 4 | 0.5 | 4 |
| **20** | TR_34_/L98H | 0.25 | >16 | 4 | 0.5 | 8 |
| **21** | TR_34_/L98H | 1 | >16 | 4 | 0.5 | >8 |
| **22** | TR_34_/L98H | 1 | >16 | 4 | 0.5 | 8 |
| **23** | TR_34_/L98H | 0.5 | >16 | 8 | 0.5 | 8 |
| **24** | TR_34_/L98H | 0.5 | >16 | 2 | 0.25 | 4 |
| **25** | TR_34_/L98H | 1 | >16 | 8 | 1 | 8 |
| **26** | TR_34_/L98H | 1 | >16 | 4 | 1 | 8 |
| **27** | TR_34_/L98H | 0.5 | >16 | 4 | 0.5 | 4 |
| **28** | TR_34_/L98H | 1 | >16 | 4 | 0.5 | 8 |
| **29** | TR_34_/L98H | 0.5 | >16 | 4 | 0.5 | 4 |
| **30** | TR_34_/L98H | 1 | >16 | 4 | 0.5 | 8 |
| **31** | TR_34_/L98H | 0.5 | >16 | 2 | 0.25 | 2 |
| **32** | TR_34_/L98H | 1 | >16 | 4 | 1 | 8 |
| **33** | TR_34_/L98H | 1 | >16 | 4 | 0.5 | 4 |
| **34** | TR_34_/L98H | 1 | >16 | 4 | 0.5 | 4 |
| **35** | TR_34_/L98H | 1 | >16 | 2 | 0.5 | 2 |
| **36** | TR_34_/L98H | 0.5 | >16 | 2 | 0.25 | 2 |
| **37** | TR_34_/L98H | 1 | >16 | 4 | 0.5 | 4 |
| **38** | TR_34_/L98H | 0.5 | >16 | 4 | 0.5 | 8 |
| **39** | TR_34_/L98H | 1 | >16 | 2 | 0.5 | 2 |
| **40** | TR_34_/L98H | 1 | >16 | 4 | 0.5 | 8 |
| **41** | TR_34_/L98H | 1 | >16 | 4 | 0.5 | 4 |
| **42** | TR_34_/L98H | 1 | >16 | 4 | 0.5 | 8 |
| **43** | TR_34_/L98H | 1 | >16 | 4 | 0.5 | 8 |
| **44** | TR_34_/L98H | 0.5 | >16 | 2 | 0.5 | 8 |
| **45** | TR_34_/L98H | 0.25 | >16 | 8 | 0.5 | 4 |
| **46** | TR_34_/L98H | 0.25 | >16 | 2 | 0.25 | 4 |
| **47** | TR_34_/L98H | 0.5 | >16 | 4 | 0.25 | 4 |
| **48** | TR_34_/L98H | 1 | >16 | 8 | 1 | 8 |
| **49** | TR_34_/L98H | 0.25 | >16 | 4 | 0.25 | 8 |
| **50** | TR_34_/L98H | 0.25 | >16 | 2 | 0.5 | 8 |
| **51** | TR_34_/L98H | 1 | >16 | 4 | 0.5 | 4 |
| **52** | TR_34_/L98H | 1 | >16 | 2 | 0.5 | 4 |
| **53** | TR_34_/L98H | 0.5 | >16 | 8 | 0.5 | 8 |
| **54** | TR_34_/L98H | 0.5 | 16 | 4 | 0.5 | 8 |
| **55** | TR_34_/L98H | 1 | 4 | 4 | 0.5 | 4 |
| **56** | TR_34_/L98H | 0.25 | >16 | 4 | 0.5 | 4 |
| **57** | TR_34_/L98H | 0.5 | 2 | 2 | 0.25 | 4 |

| **N** | **CYP51A** | **AMB** | **ITC** | **VRC** | **POS** | **ISA** |
| --- | --- | --- | --- | --- | --- | --- |
| **1** | TR_46_/Y121F/T289A | 0.25 | 0.5 | 16 | 0.125 | >16 |
| **2** | TR_46_/Y121F/T289A | 0.25 | 0.5 | >16 | 0.125 | >16 |
| **3** | TR_46_/Y121F/T289A | 0.25 | 0.5 | >16 | 0.25 | >16 |
| **4** | TR_46_/Y121F/T289A | 0.25 | 0.5 | >16 | 0.125 | >16 |
| **5** | TR_46_/Y121F/T289A | 0.5 | 1 | 16 | 0.125 | >16 |
| **6** | TR_46_/Y121F/T289A | 1 | >16 | >16 | 0.5 | >8 |
| **7** | TR_46_/Y121F/T289A | 1 | >16 | >16 | 1 | >8 |
| **8** | TR_46_/Y121F/T289A | 0.5 | >16 | >16 | 0.5 | >8 |
| **9** | TR_46_/Y121F/T289A | 1 | 2 | >16 | 0.5 | >8 |
| **10** | TR_46_/Y121F/T289A | 1 | 2 | >16 | 1 | >8 |
| **11** | TR_46_/Y121F/T289A | 0.5 | >16 | >16 | 1 | >8 |
| **12** | TR_46_/Y121F/T289A | 1 | >16 | >16 | 1 | >8 |
| **13** | TR_46_/Y121F/T289A | 1 | 2 | >16 | 0.5 | >8 |
| **14** | TR_46_/Y121F/T289A | 0.5 | >16 | >16 | 1 | >8 |
| **15** | TR_46_/Y121F/T289A | 1 | >16 | >16 | 0.5 | 8 |
| **16** | TR_46_/Y121F/T289A | 1 | 0.5 | >16 | 0.25 | >8 |
| **17** | TR_46_/Y121F/T289A | 0.5 | 2 | >16 | 0.5 | >8 |
| **18** | TR_46_/Y121F/T289A | 0.125 | >16 | 16 | 1 | >16 |
| **19** | TR_46_/Y121F/T289A | 1 | >16 | >16 | 1 | >16 |
| **20** | TR_46_/Y121F/T289A | 0.25 | 1 | 16 | 0.5 | >16 |
| **21** | TR_46_/Y121F/T289A | 0.25 | 1 | 16 | 1 | >16 |
| **22** | TR_46_/Y121F/T289A | 0.25 | 2 | 16 | 1 | >16 |
| **23** | TR_46_/Y121F/T289A | 0.25 | >16 | 16 | 1 | >16 |
| **24** | TR_46_/Y121F/T289A | 0.25 | 4 | 16 | 1 | >16 |
| **25** | TR_46_/Y121F/T289A | 0.5 | 0.25 | 16 | 0.25 | >16 |
| **26** | TR_46_/Y121F/T289A | 0.5 | 1 | 16 | 0.5 | >16 |
| **27** | TR_46_/Y121F/T289A | 0.5 | 0.25 | 8 | 0.125 | >8 |
| **28** | TR_46_/Y121F/T289A | 1 | 2 | >16 | 0.5 | >16 |
| **29** | TR_46_/Y121F/T289A | 0.5 | >16 | 16 | >8 | >16 |
| **30** | TR_46_/Y121F/T289A | 1 | >16 | 16 | 1 | >16 |
| **31** | TR_46_/Y121F/T289A | 1 | 1 | 16 | 0.5 | >16 |
| **32** | TR_46_/Y121F/T289A | 0.5 | 2 | >16 | 0.5 | >16 |
| **33** | TR_46_/Y121F/T289A | 1 | 4 | >16 | 0.5 | 1 |
| **34** | TR_46_/Y121F/T289A | 0.5 | 2 | 16 | 0.5 | >16 |
| **35** | TR_46_/Y121F/T289A | 0.5 | 0.5 | 16 | 0.25 | >8 |
| **36** | TR_46_/Y121F/T289A | 0.5 | 1 | 16 | 0.5 | >16 |
| **37** | TR_46_/Y121F/T289A | 0.25 | 2 | 16 | 1 | >16 |
| **38** | TR_46_/Y121F/T289A | 1 | >16 | >16 | 1 | >16 |
| **39** | TR_46_/Y121F/T289A | 1 | 4 | 8 | 1 | >8 |
| **40** | TR_46_/Y121F/T289A | 1 | >16 | >16 | 0.5 | >16 |
| **41** | TR_46_/Y121F/T289A | 1 | >16 | >16 | 2 | >16 |
| **42** | TR_46_/Y121F/T289A | 1 | >16 | >16 | 1 | >16 |
| **43** | TR_46_/Y121F/T289A | 0.5 | >16 | >16 | 1 | >16 |
| **44** | TR_46_/Y121F/T289A | 1 | >16 | >16 | 1 | >16 |
| **45** | TR_46_/Y121F/T289A | 1 | >16 | >16 | 1 | >16 |
| **46** | TR_46_/Y121F/T289A | 0.5 | 2 | >16 | 1 | >16 |
| **47** | TR_46_/Y121F/T289A | 0.25 | 2 | >16 | 0.5 | >16 |
| **48** | TR_46_/Y121F/T289A | 0.25 | >16 | >16 | 1 | >16 |
| **49** | TR_46_/Y121F/T289A | 0.5 | 0.25 | 0.5 | 0.0>8 | 0.5 |
| **50** | TR_46_/Y121F/T289A | 0.25 | 1 | >16 | 0.25 | >16 |
| **51** | TR_46_/Y121F/T289A | 1 | >16 | >16 | 1 | >16 |
| **52** | TR_46_/Y121F/T289A | 0.5 | 1 | >16 | 0.5 | >16 |
| **53** | TR_46_/Y121F/T289A | 0.25 | >16 | >16 | 0.5 | >16 |
| **54** | TR_46_/Y121F/T289A | 1 | >16 | >16 | 0.25 | >16 |

| **N** | **CYP51A** | **AMB** | **ITC** | **VRC** | **POS** | **ISA** |
| --- | --- | --- | --- | --- | --- | --- |
| **1** | G54R | 0.25 | >4 | 0.5 | 4 | 0.5 |
| **2** | G54R | 0.25 | >16 | 0.25 | 4 | 0.5 |
| **3** | P216S | 2 | >16 | 1 | 0.5 | 2 |
| **4** | P216S | 2 | 0.5 | 1 | 0.125 | 1 |
| **5** | G448S | 0.5 | >16 | 8 | 0.25 | 16 |
| **6** | G448S | 1 | >16 | 16 | 0.25 | 16 |
| **7** | M220K | 0.25 | >16 | 1 | 1 | 1 |
| **8** | M220I | 0.5 | >16 | 1 | 0.5 | 2 |
| **9** | F219S | 0.25 | 16 | 0.5 | 0.25 | 1 |
| **10** | G448S | 0.5 | >4 | >4 | 1 | >8 |
| **11** | G432S | 1 | >16 | 4 | 2 | 16 |
| **12** | M220R | 0.5 | >16 | 2 | >4 | 2 |
| **13** | M220R | 0.5 | >16 | 4 | >4 | 8 |
| **14** | M220R | 0.5 | >16 | 1 | 1 | 2 |
| **15** | G54R | 0.5 | >4 | 1 | >4 | 1 |
| **16** | TR120/F46Y/M172V/E427K | 0.25 | >16 | 4 | 0.5 | 4 |
| **17** | G54R | 0.5 | >4 | 1 | >4 | 1 |
| **18** | G54W | 0.5 | >16 | 0.5 | >4 | 0.5 |
| **19** | G54R | 0.5 | >16 | 8 | >4 | 4 |
| **20** | G54R | 0.125 | >16 | 8 | >4 | 16 |
| **21** | M220K | 0.25 | >4 | 2 | 2 | 2 |
| **22** | M220K | 0.25 | >4 | 1 | 2 | 2 |
| **23** | M220K | 0.25 | >4 | 2 | >4 | 2 |
| **24** | M220K | 0.25 | >4 | 1 | 1 | 1 |
| **25** | Y121F | 0.125 | >16 | >16 | 1 | >8 |
| **26** | Y121F | 1 | >16 | >16 | 2 | >16 |
| **27** | P216S | 2 | 0.5 | 1 | 0.125 | 1 |

AMB=amphotericin B, ITC=itraconazole, VRC=voriconazole, POS=posaconazole, ISA=isavuconazole
